# Transmission of Epi-Alleles with MET1-Dependent Dense Methylation in *Arabidopsis thaliana*

**DOI:** 10.1371/journal.pone.0105338

**Published:** 2014-08-19

**Authors:** Michael Watson, Emily Hawkes, Peter Meyer

**Affiliations:** Center for Plant Sciences, University of Leeds, Leeds, United Kingdom; University of Guelph, Canada

## Abstract

DNA methylation in plants targets cytosines in three sequence contexts, CG, CHG and CHH (H representing A, C or T). Each of these patterns has traditionally been associated with distinct DNA methylation pathways with CHH methylation being controlled by the RNA dependent DNA methylation (RdDM) pathway employing small RNAs as a guide for the *de novo* DOMAINS REARRANGED METHYLTRANSFERASE (DRM2), and maintenance DNA METHYLTRANSFERASE1 (MET1) being responsible for faithful propagation of CG methylation. Here we report an unusual ‘dense methylation’ pattern under the control of MET1, with methylation in all three sequence contexts. We identified epi-alleles of dense methylation at a non coding RNA locus (*At4g15242*) in *Arabidopsis* ecotypes, with distinct dense methylation and expression characteristics, which are stably maintained and transmitted in genetic crosses and which can be heritably altered by depletion of MET1. This suggests that, in addition to its classical CG maintenance function, at certain loci MET1 plays a role in creating transcriptional diversity based on the generation of independent epi-alleles. Database inspection identified several other loci with MET1-dependent dense methylation patterns. *Arabidopsis* ecotypes contain distinct epi-alleles of these loci with expression patterns that inversely correlate with methylation density, predominantly within the transcribed region. In *Arabidopsis*, dense methylation appears to be an exception as it is only found at a small number of loci. Its presence does, however, highlight the potential for MET1 as a contributor to epigenetic diversity, and it will be interesting to investigate the representation of dense methylation in other plant species.

## Introduction

DNA methylation patterns in plants influence a number of molecular mechanisms, including transcription [1], repair [2] and recombination [3], with implications for plant development [4], genome structure [5] and evolution [6]. The epi-genotype has therefore emerged as an additional factor to genetic mutations in shaping phenotypic diversity [7], [8]. A remarkable example for the evolutionary role of epigenetic traits comes from the analysis of populations of the mangrove *L. racemosa* with plants collected from salt marsh and riverside locations, respectively, showing little genetic variation but substantial differences in DNA methylation marks [9]. The responsiveness of DNA methylation patterns to environmental stress [10] has been suggested to act as a molecular switch for evolutionary adaptation of plants to environmental change [11]. In support of this model, various biotic [12] and abiotic stress conditions [13] have been shown to alter DNA methylation profiles.

Cytosine methylation in *Arabidopsis* occurs in three sequence contexts. The most prominent methylation mark at CG sites is faithfully propagated by maintenance DNA METHYLTRANSFERASE1 (MET1), a plant homolog of the mammalian DNA methyltransferase 1 (DNMT1). Non-symmetrical CHH methylation is controlled by the RNA-directed DNA methylation (RdDM) pathway with 24nt small RNAs (siRNAs) as guides for *de novo* DOMAINS REARRANGED METHYLTRANSFERASE 2 (DRM2) and its weakly active homolog DRM1. The RdDM pathway predominantly targets repeats in heterochromatic regions, and in dispersed transposons and related sequences in euchromatic regions [14]. While DRM1 and DRM2 are homologs of mammalian *de novo* methyltransferase DNMT3, the third DNA methyltransferase, CHROMOMETHYLASE3 (CMT3), is exclusively found in plants. CMT3 predominantly controls CHG methylation [15] in combination with three histone methyltransferases, the SU(VAR)3-9 homologues SUVH4, SUVH5 and SUVH6. CMT3 and DRM1/2 act redundantly to maintain CHG and CHH methylation marks [16], which co-localise in *Arabidopsis* [17].

The analysis of distinct genomic loci has helped to establish mechanistic models that allocated specific functions to the different DNA methyltransferases. MET1 has mainly been discussed in the context of its maintenance function for CG methylation marks, providing more stable epigenetic patterns than the target loci of the RdDM pathway, which show a higher level of epigenetic variation in *Arabidopsis* accessions [18]. Variations in siRNA frequencies and cytosine methylation have also been found at RdDM target loci in hybrid plants due to Trans Chromosomal Methylation and Demethylation (TCM/TCdM) effects. Hybrid-specific epigenetic changes can be heritable over at least one generation and can alter gene expression levels, thus providing an attractive model for the involvement of the RdDM pathway in heterosis [19]

MET1 has a crucial role in maintenance of CG methylation, an essential regulator of *trans*-generational inheritance of epigenetic patterns. MET1 elimination is deleterious for *Arabidopsis*, which only tolerates reductions in MET1 levels. Plants homozygous for the *met1-3* null allele of MET1, which have lost MET1 activity completely, only survive due to random changes in DNA demethylation and *de novo* methylation functions [20]. MET1 therefore has at least an indirect role in epigenetic variation due to the responsiveness of the RdDM pathway to changes in CG methylation levels. Another indirect effect on non-CG methylation has been observed at certain loci that lose their H3K9 methylation patterns in a *met1* mutant, which resulted in a loss of CHG and CHH methylation marks [21]. While loss of CHG methylation at such loci in a *met1* mutant can be explained by a loss of chromatin marks required for CMT3 binding, it remains unclear if loss of CHH marks reflects a failed interaction between CMT3 and DRM2, or a direct role of MET1 in CHH methylation targeting.

Here we report a controlling role for MET1 in non-CG methylation for a small number of loci with a dense methylation pattern in all three sequence contexts. Dense methylation is MET1-dependent but independent of DRM1/2 activity, and predominantly extends throughout transcribed regions of affected genes, inversely correlating with transcript levels.

## Results

### MET1-dependent, ecotype-specific alleles of a ncRNA locus

**Figure 1:**
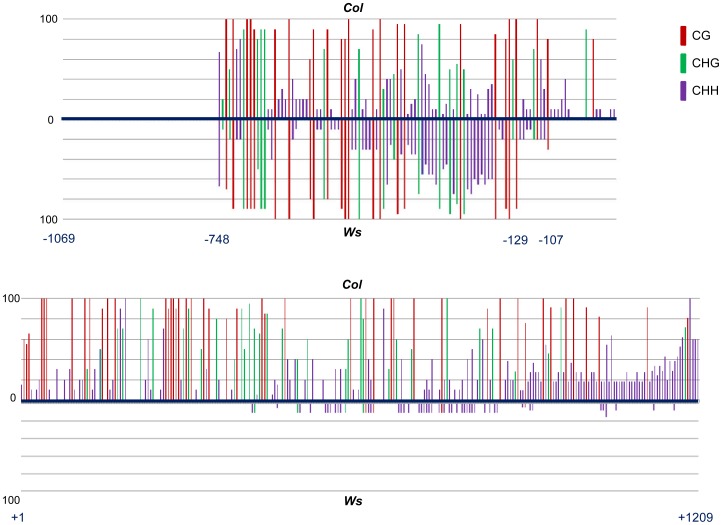
DNA methylation in the silenced *At4g15242* allele in ecotype *Columbia (Col)* and the active *At4g15242* epi-allele in ecotype *Wassilewskija (Ws)* in promoter (top panel) and transcribed region (lower panel). Polymorphic regions were removed to show only sites where both accessions have a C in the same sequence context. Both epi-alleles share a similar methylation profile in the upstream promoter region (-748 to -129), while in *Ws* methylation is almost completely eliminated 107nt upstream of the transcription start site and within the transcribed region.

When investigating the function of a non-coding RNA locus, *At4g15242*, we noticed that the gene was expressed in *Arabidopsis* ecotype *Wassilewskija (Ws)* but not in ecotype *Columbia (Col)*. Bisulphite sequencing analysis revealed a dense DNA methylation pattern in all sequence contexts of the locus in both ecotypes. In the inactive *Col* allele, methylation covered a 748bp promoter region and the complete 1209bp transcribed region, while in the active *Ws* allele, methylation was restricted to almost the complete promoter region but almost fully absent in the transcribed region and within a 3′ promoter region 107bp upstream of the transcription start site (Figure 1). This suggested a link between the differential expression of the two alleles and differences in the extension of the dense methylation pattern. Within the 2278bp region, *Col* and *Ws* alleles differ at 34 polymorphic positions (Figure S1), which may also influence allelic expression states.

**Figure 2:**
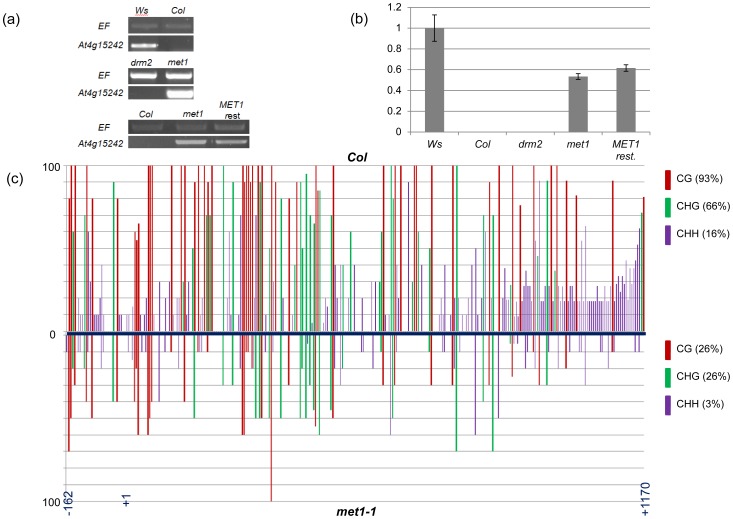
Expression and methylation patterns of *At4g15242*. (a)Semi-quantitative RT-PCR analysis of *At4g15242* expression in *Ws*, *Col* and in *Col* derived mutants *drm2*, *met1-1* and for a line, which derived from a backcross of *met1* with wildtype, and which contains two wildtype *MET1* alleles (MET*1 rest.*). (b)Quantitative RT-PCR values were compared to a reference value of 1 for *Ws*, and are shown for *Col*, *Col* derived mutants *drm2* and *met1*, and for MET*1 rest*. Activation of *At4g15242* in *met1* is not reversed when *MET1* alleles are introduced. (c)Comparison of DNA methylation levels in the -162 to +1170 region of *At4g15242* in *Col* and, *met1-1*.

To examine which DNA methylation pathway contributed to the dense methylation pattern and if changes in DNA methylation were causal for the activation of expression, we tested the expression of *At4g15242* in a *met1* mutant and a *drm2* mutant, both in a *Columbia* background. Expression was not affected by loss of DRM2 but the gene was activated in a *met1-1* mutant to about half the expression level observed in *Wassilewskija* (Figure 2a and 2b). As *met1-1* is not a null-mutant it does not completely eliminate DNA methylation. Accordingly, methylation of *At4g15242* in a *met1-1* mutant is reduced to about 30% of the methylation levels found in Columbia wildtype (Figure 2c), resembling an intermediate methylation state between the methylation states of the *At4g15242* epi-alleles in *Columbia* and *Wassilewskija*. Our data implied that the dense methylation pattern of *At4g15242* is controlled by MET1 and that methylation intensity and transcript levels are linked. It is unclear if differences in overall methylation levels or at specific cytosines are responsible for changes in expression. To examine if an activated *At4g15242* allele remained active or if silencing was re-established once MET1 levels were restored, we self-pollinated a plant derived from a cross between the *met1-1* mutant and a *Columbia* wildtype line, and selected a line homozygous for the wildtype MET1 alleles. The activated *At4g15242* allele remained expressed in this line (Figure 2b), suggesting that MET1 depletion caused heritable activation that was stable over at least two generations.

### At4g15242 epi-alleles are independently transmitted in genetic crosses

**Figure 3:**
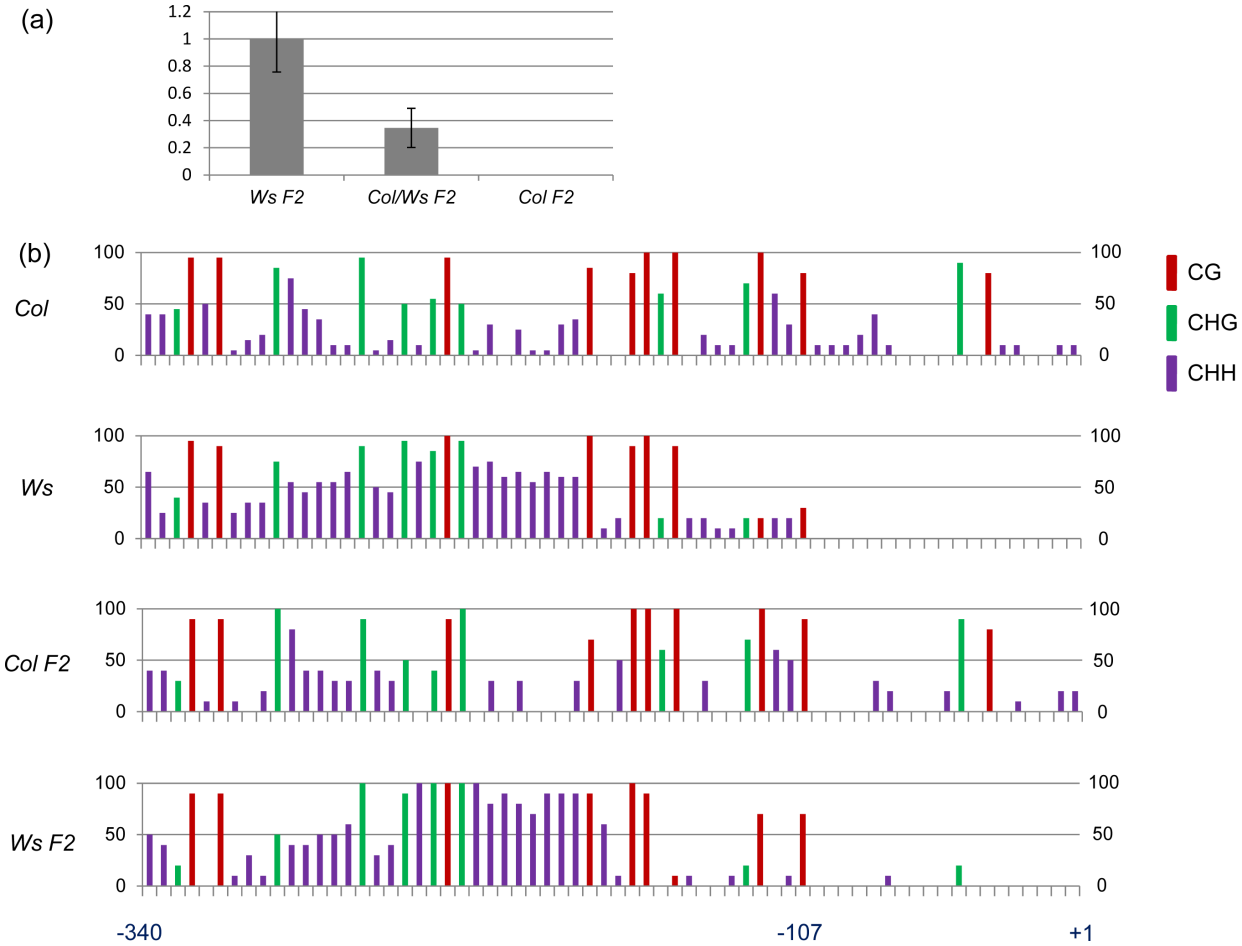
Stability of expression and methylation patterns of *At4g15242 Col* and *Ws* alleles. (a)Comparison of *At4g15242* expression in F2 plants derived from a *Col/Ws* hybrid. Plants contain two *Wassilewskija* epi-alleles (*Ws* F2), one *Wassilewskija* and one *Columbia* epi-allele (*Col/Ws* F2) or two *Columbia* epi-alleles (*Col* F2). Quantitative RT-PCR values were based on data from three different plants and were compared to a reference value of 1 for *Ws* F2. (b)Methylation profile in the -340 to +1 promoter region of *Col* and *Ws* epi-alleles before and after combination passage through a *Col/Ws* F1 hybrid. Both alleles retain their specific methylation patterns.

If the activated *At4g15242* allele remained stably active, it was conceivable that *At4g15242* alleles could also be maintained and propagated as independent, stable epi-alleles when combined in genetic crosses. To test this assumption, we followed expression and methylation of *Col* and *Ws* epi-alleles of *At4g15242* in genetic crosses. Expression analysis was based on RT-PCR data from three F2 lines that were used as biological replicas. Genomic DNA samples from three F2 plants were pooled for DNA methylation analysis. A sequence polymorphism helped to follow the origin of the alleles in F2 lines derived from self-pollination of a *Col/Ws* F1 hybrid line. As in the *Columbia* progenitor, *At4g15242* was silenced in F2 plants with two *Col* alleles, while in F2 plants with two *Ws* alleles these were expressed, and *Col/Ws* F2 hybrids showed an intermediate *At4g15242* expression level (Figure 3a). A similar conservation was observed for the *Col* and *Ws* methylation patterns in F2 lines. Comparison of the *Col* and *Ws* alleles had identified the region upstream of position -129 as hypermethylated in both alleles, and the region downstream of position -107 to be hypermethlayed in *Col* but hypomethylated in *Ws*. The same methylation characteristics were retained in the *Col* and *Ws* F2 population. Both F2 alleles were hypermethylated upstream of position -129. Downstream of position -107, *Col*–specific hypermethylation and *Ws*-specific hypomethylation was retained in the F2 alleles (Figure 3b), which suggests that both epi-alleles independently retain their expression and methylation patterns in genetic crosses.

### Dense methylation patterns are not restricted to At4g15242

**Figure 4:**
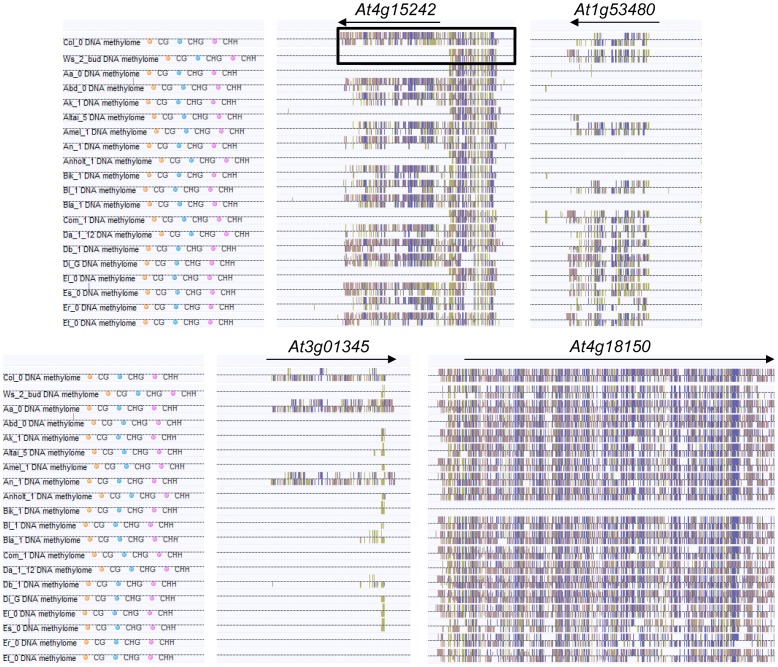
DNA methylation patterns of *At4g15242* and three other genes with MET1-dependent dense methylation in different *Arabidopsis* ecotypes [18], extracted from http://neomorph.salk.edu/1001_epigenomes.html. The box marks the methylation patterns of *At4g15242* in *Col* and *Ws* alleles, which matches the bisulphite analysis shown in figure 1. For each gene, methylation levels are marked for cytosines in CG (yellow), CHG (blue) and CHH (red) sequence contexts.

**Figure 5:**
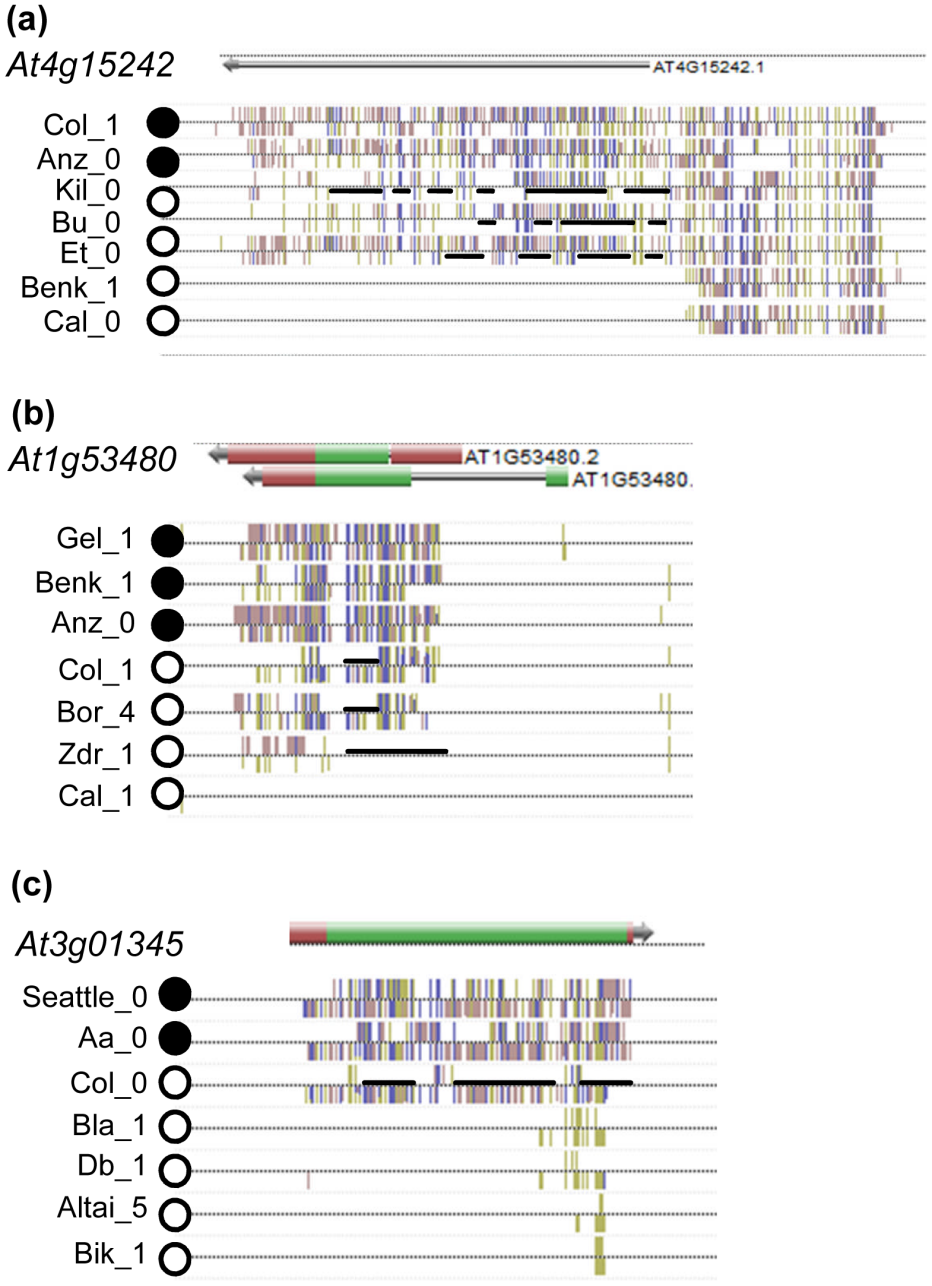
Comparison of DNA methylation patterns in active (open circle) and repressed alleles (filled circles) of three genes with MET1-dependent dense methylation extracted from http://neomorph.salk.edu/1001_epigenomes.html. Black lines label strand-specific methylation-free regions in active alleles.

To investigate if the MET1-dependent dense methylation pattern we had detected in *At4g15242* was present at other loci, especially in genes with protein coding information, we inspected a database that had become publically available during our studies, which contains DNA methylation data for 86 epigenetic mutants [21]. By manual inspection of the methylome database, we selected three coding genes with dense methylation in CG, CHG and CHH contexts, predominantly within their transcribed regions. According to the Arabidopsis Information Resource (TAIR) database [22], *At1g53480* encodes an unknown protein, *At3g01345* a protein with hydrolase activity and *At4g18150* a kinase-related protein. As for *At4g15242*, dense methylation in the three genes was retained in a *drm1/2* mutant but eliminated in a *met1* mutant (Figure S2). All but one gene retained their dense methylation pattern in *ago4, dcl2/3/4, nrpd1, nrpe1* and *rdr2* mutants (Figure S3), indicative for their independence from the RdDM pathway. Another common feature of all four lines was the dependence of CHG methylation marks on CMT3 and of CHH methylation marks on CMT2 (Figure S4). A screen of methylation and expression profiles for different *Arabidopsis* accessions [18], revealed epi-alleles of all four genes represented among ecotypes (Figure 4). *At4g18150* epi-alleles were either fully methylated or unmethylated but for the other three genes, epi-alleles with different methylation levels could be found. Alignment of these methylation patterns for expressed and silenced epi-alleles showed a potential correlation between transcriptional activation and reduction in methylation density within transcribed regions (Figure 5).

## Discussion

We identified an unusual dense DNA methylation pattern in four genes, which affects cytosines in all sequence contexts. As methylation in all sequence contexts is lost in a *met1* mutant, we labelled this pattern MET1 dependent Dense Methylation (MdDM). MdDM is independent of the RdDM pathway methyltransferase DRM1/2 but requires CMT2 for maintenance of CHH methylation marks. *Arabidopsis* accessions contain distinct epi-alleles of the four dense methylation genes with expression levels that correlate inversely with the overall density of methylation within the transcribed region. Without being able to exclude the influence of a specific sequence element that is only present in one allele, or the methylation of a defined cytosine on allelic expression differences, we favour the option of an inverse link between expression and overall density of DNA methylation. In contrast to epi-alleles that regulate gene silencing via hypermethylation of promoter regions [23]; [24]; [25]; [26] our data suggest that expression of genes with MdDM is controlled by methylation density within the transcribed regions. In this respect, dense methylation resembles methylation patterns in transcribed regions of the cysteine-rich peptides 4 (*CRP4*) and other members of the *CRP* family [27]. In contrast to MdDM, methylation of *CRP*4 is controlled by the RdDM pathway and not eliminated in a *met1* mutant background. It has been suggested that the *CRP* family has evolved through retrotransposition, retaining methylation patterns characteristic for transposons controlled by the RdDM pathway [27]. Equally, it is conceivable that genes with MdDM have evolved from retrotransposons with RdDM-independent DNA methylation features. RdDM-independent transposon methylation is mediated by the nucleosome remodeler DDM1 with CHH methylation depending on CMT2 [28]. Accordingly, MdDM shares some similarities with the DDM1-dependent methylation patterns of *Gypsy* elements, which also carry DRM2-independent CHH methylation marks that require CMT2 and CHG marks, which are at least partially affected in a *cmt3* mutant background. In contrast to the four MdDM genes, *Gypsy* elements are not activated in a *met1* mutant, and respond in different ways to MET1 depletion as some elements are fully demethylated while others remain unaffected or only lose CG methylation marks (Figure S5a). In *Arabidopsis* ecotypes, we find a similar variation in epi-alleles for *Gypsy* elements as observed for the four MdDM genes (Figure S5b). Hypomethylated epi-alleles of *Gypsy* elements are, however, not transcribed, probably because they are under additional control by the RdDM pathway at their edges [28].

An interesting feature of MdDM is the dependence of DNA methylation marks in all sequence contexts on MET1, as depletion of MET1 leads to hypomethylation of cytosines in all sequence contexts. MET1 is therefore not only required for maintenance of CG methylation but also acts as a coordinator for CMT2- and CMT3-mediated methylation of CHH and CHG sites. Epi-alleles of the ncRNA locus *At4g15242* qualify as ‘pure epi-alleles’ [29] as both the active and inactive alleles retain their methylation and expression profiles and segregate independently in genetic crosses, at least over two generations. We can, however, not exclude that activated epi-alleles become remethylated over several generations, similar to some loci that are hypomethylated in a *ddm1* mutation, and which very slowly restore their methylation pattern, while others remain hypomethylated [30]. The variation in MdDM epi-alleles with distinct methylation and expression patterns in *Arabidopsis* ecotypes supports the assumption that various epi-alleles have been generated during evolution that are maintained in individual ecotypes. The independence of dense methylation from the RdDM pathway most likely contributes to the stability and autonomy of MdDM epi-alleles. SiRNAs are responsible for the restoration of RNAi dependent methylation at repeats as a protection against transgenerational loss of methylation [31]. At least at some loci, siRNAs mediate Trans Chromosomal Methylation/deMethylation effects in hybrid epigenomes [19].

Expression of the four MdDM genes appears to inversely correlate with methylation density, predominantly in transcribed regions. Epigenetic control of transcription is often associated with methylation changes in 5′ or promoter regions [23]; [24]; [25]; [26] but methylation changes can extend into the transcribed region, and critical methylation target regions have either not been defined [32]; [33] or have been allocated to transcribed regions [27]. While it can't be excluded that expression of MdDM target genes is controlled by defined cytosine, we consider it more likely that methylation density prevents access of the transcription machinery to the inactive epi-alleles due to formation of a heterochromatic state, which can switch to a stable, accessible state when methylation density is reduced below a specific threshold. We favour a model that dense methylation prevents initiation of transcription but alternatively, dense methylation within transcribed regions may allow initiation of transcription but prevent transcript elongation.

While MdDM epi-alleles behave autonomously, they are dependent on CMT2 to maintain their CHH methylation marks and especially on MET1 as a co-ordinator of all methylation types. It remains to be investigated how MET1 coordinates methylation in all sequence context. A useful model could be the loss of histone methylation marks that can be induced by depletion of CG methylation at heterochromatic loci [34]. MET1 controlled CG methylation may induce histone modifications at MdDM target loci, which are required to recruit CMT2 and CMT3.

*At4g15242* shows a very sensitive response to changes in MET1 functionality as its transcription is already activated when local methylation levels are reduced by about two thirds in the *met1-1* mutant. Once transcription has been activated the epi-allele is not silenced again when a functional MET1 activity is re-introduced. A reduction of DNA methylation levels by two thirds is therefore sufficient to cause stable and heritable activation of *At4g15242*. As the ncRNA locus remains repressed in the *Col* wildtype, this argues against models that propose significant changes in MET1 activity in gametes [35] and in favour of models supporting a stable MET1 activity [36] as it would be required to secure the repressive states of the silent *At4g15242 Col* epi-allele. The sensitivity of MdDM target loci to MET1 activity may, however, be the basis for epi-allelic changes due to temporary changes in MET1 concentration or efficiency, providing a potential link between changing environmental conditions and a change in epigenetic states that would contribute to natural epigenetic variation as previously suggested [37].

In *Arabidopsis*, MdDM only affects a few genes but it will be interesting to investigate if MdDM targets are more widely represented in other species, especially in those that show a significant increase of non-CG methylation in transcribed regions [38]. For species with a higher number of MdDM targets controlled down-regulation of MET1 functions could become a promising strategy to enhance heritable epigenetic diversity.

## Experimental Procedures

### Plant material and genotyping of alleles

The *ddm2-1/met1-1* (*At5g49160*) mutant in *Columbia* ecotype background was a kind gift from Dr. Mittelsten Scheid. The *drm2-2* (*At5g14620*) DNA insertion line (SALK_150863) in *Columbia* ecotype background was obtained from the Nottingham Arabidopsis Stock Centre (http://arabidopsis.info). All plants were grown in a growth chamber under long day conditions (16 hours light, 8 hours dark, 22°C temperature and 60% humidity) unless stated otherwise. Genomic DNA for genotyping was extracted from 2-week old seedlings according to [39]. PCR reactions were performed using the MyTaq Red DNA polymerase (Bioline) following the manufacturer's recommendation. A SNP at position ch.4, 8707153 bp (*Col* sequence T, *Ws* sequence C) was used to distinguish between *Columbia* and *Wassilewskija* alleles of *At4g15242*.

### Expression and DNA methylation analysis

Total RNA was extracted as described [40] from a pool of ten 2-week old seedlings, except for F2 lines, where total RNA was isolated from leaves of individual 4-week old plants. RNA was treated with DNase (Ambion, Austin, TX) and cDNA synthesis was performed on 2µg of RNA using Superscript II Reverse Transcriptase (Invitrogen, Paisley, UK) and random primers (Promega) according to the manufacturer's recommendation.

List of forward and reverse primers used for RT-PCR analysis:

At4g15242: forward 5′ CGATCTGTGCGCTTTACTCCC, reverse 5′ GGCTTGGGAAATGGAAAGAGG

EF1a (AT1G07940): forward 5′ CTCTCCTTGAGGCTCTTGACCAG, reverse 5′ CCAATACCACCAATCTTGTAGACATCC

Genomic DNA was isolated from a pool of ten 2-week old seedlings, except for F2 lines, where genomic DNA was isolated from leaves of individual 4-week old plants. Genomic DNA was isolated [41] and subjected to bisulfite treatment using an EZ DNA Methylation-lightning kit (Zymo Research) according to the manufacturer's instructions. Eight fragments (A-H) were amplified to analyze the methylation pattern of the *At4g15242* region between ch.4, 8,708,066 and ch.4, 8,705,797. For each line, 10 clones were sequenced and sequences were exported into the BioEdit program [42]. Aligned sequences were saved in FASTA format and analyzed by the CyMATE programme [43].

List of forward and reverse primers used for bisulphite sequencing:

Fragment A: forward 5′ TGATTAYAATTATTAAAGATTATGTGA, reverse 5′ ATTTATAAATARTAAATAAAAATTCA

Fragment B: forward 5′ TATTTATAAATTGTGTATTGTAAG, reverse 5′ CATCATTAAATATATCATTTAAAC

Fragment C: forward 5′ AAATTTATGATATAYTGATAAAATTA, reverse 5′ TTTACCATTCATAARCTATAATCC

Fragment D: forward 5′ AGTTTATGTTTTAGGTTTTGAATGAATG, reverse 5′ CCACRCACRTCRACTTCTTCTTTT

Fragment E: forward 5′ AAAAGAAGAAGTYGAYGTGYGTGG, reverse 5′ CACCAAAAARARACCAACTTCCCC

Fragment F: forward 5′ TGTTTGTGTGTTGYTGTTTTAGGTGTAG, reverse 5′ CCAAAARTRTTARRCAATRCTTACTCACTCTAAC

Fragment G: forward 5′ GATTGGTGGTGTTAGTATGGCTCTTTGTG, reverse 5′ TAAATATTCATTATCACAATRAAAATTTC

Fragment H: forward 5′ GAGGATTAGGTTYAAGAATGTTGTATG, reverse 5′ CACAACRAARCARTCACTTTC

## Supporting Information

Figure S1 **Sequence comparison of** ***Col*** **and** ***Ws*** **alleles of** ***At4g15242*** **region.** Boxes marks region with sequence polymorphisms.
(TIF)

Figure S2 **Methylation profile of four loci with dense DNA methylation in** ***Columbia*** **wildtype,** ***met1-3*** **,** ***drm1/2 and drm1/2/cmt3*** **mutant lines** [21] **, accessed via** http://genomes.mcdb.ucla.edu/AthBSseq/ **).** All genes contain dense methylation, predominantly within the transcribed region, in all sequence contexts, which is maintained in *drm1/2* but dependent on MET1. CMT3 depletion removes most of the CHG marks.
(TIF)

Figure S3 **Methylation profile of four loci with dense DNA methylation in** ***Columbia*** **wildtype and in RdRM pathway mutants** ***ago4, dcl2/3/4, nrpd1, nrpe1*** **and** ***rdr2*** **** [21] **, accessed via** http://genomes.mcdb.ucla.edu/AthBSseq/ **).** With the exception of *At1g53480*, none of the genes change their methylation patterns in any of the mutants.
(TIF)

Figure S4 **Methylation profile of four loci with dense DNA methylation in** ***Columbia*** **wildtype,** ***cmt2/3, cmt2, cmt3 and ddm1*** **mutant lines** [21] **, accessed via** http://genomes.mcdb.ucla.edu/AthBSseq/ **).** In all genes, CHH methylation is significantly reduced in a *cmt2* mutant. Loss of DDM1 causes loss of dense methylation in *At1g53480* and partial loss of dense methylation in the other three lines.
(TIF)

Figure S5 **Methylation profiles of** ***Gypsy*** **elements in mutants and ecotypes.** (a)Methylation profile of three *Gypsy* elements in *Columbia* wildtype, *ddm1, met1, cmt2, cmt3* and *drm1/2* mutant lines [21]. Methylation of the elements differs with respect to dependence on *ddm1* and *met1*. A common feature of all elements is their dependence on CMT2 for CHH methylation and a reduction in CHG methylation in a *cmt3* background. (b)Variable DNA methylation of the three *Gypsy* elements in different *Arabidopsis* ecotypes [18]. Irrespective of the methylation status, neither element is expressed.
(TIF)
