# Supplementary figures and images for: Transmission of Epi-Alleles with MET1-Dependent Dense Methylation in Arabidopsis thaliana
Source: PLoS One. 2014 Aug 19;9(8):e105338. doi: 10.1371/journal.pone.0105338 (PMC4138171; doi:10.1371/journal.pone.0105338)

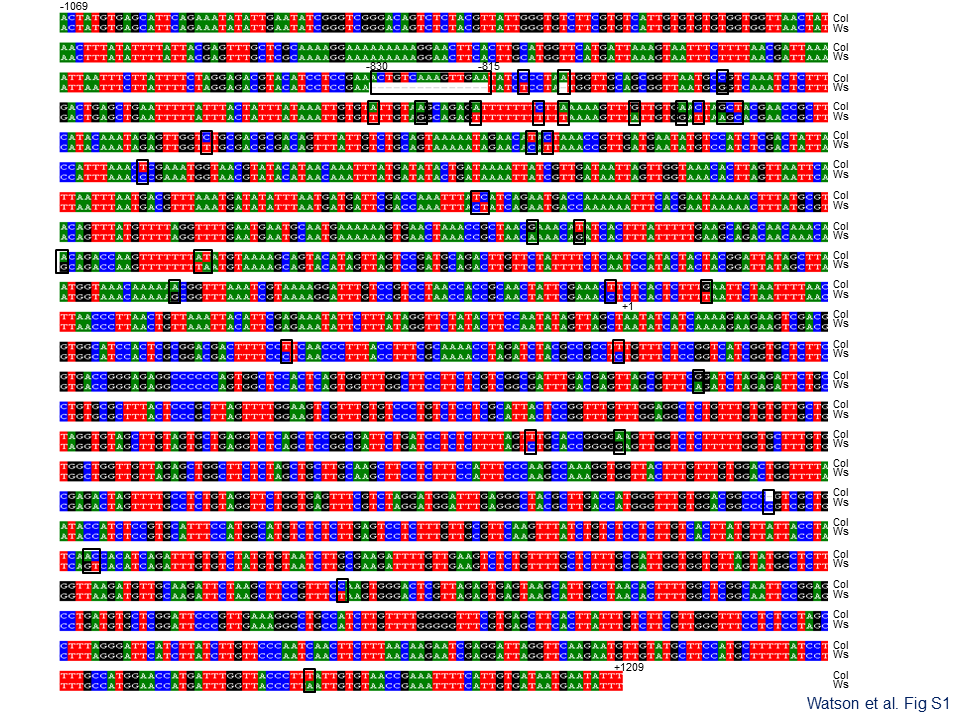

Supplement: Figure S1 — Sequence comparison of Col and Ws alleles of At4g15242 region. Boxes marks region with sequence polymorphisms. (TIF) [file pone.0105338.s001.tif]

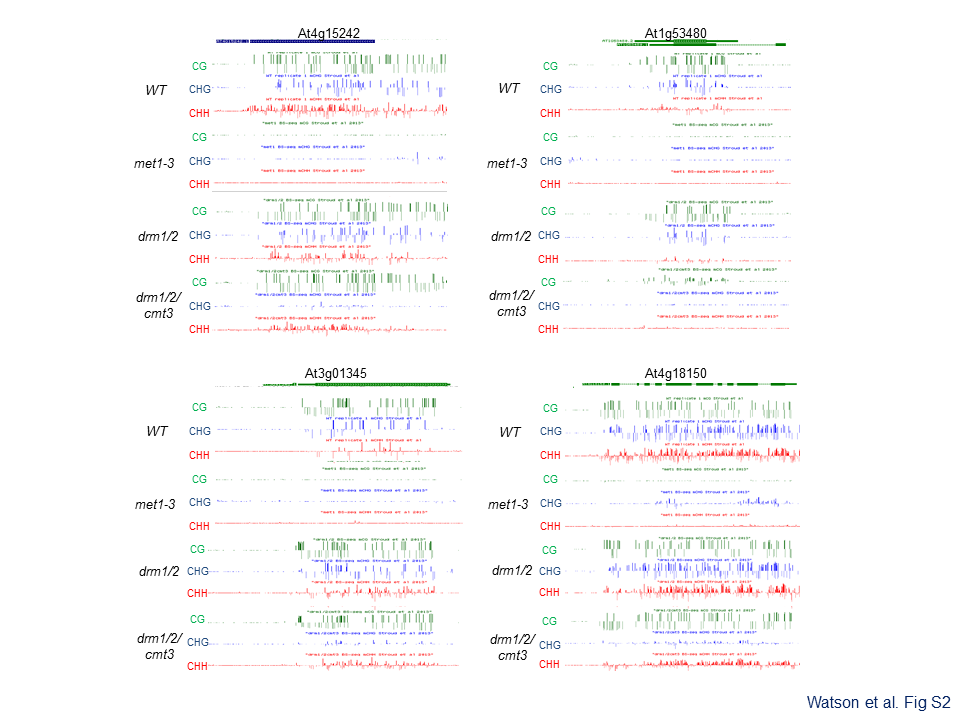

Supplement: Figure S2 — Methylation profile of four loci with dense DNA methylation in Columbia wildtype, met1-3 , drm1/2 and drm1/2/cmt3 mutant lines [21] , accessed via http://genomes.mcdb.ucla.edu/AthBSseq/ ). All genes contain dense methylation, predominantly within the transcribed region, in all sequence contexts, which is maintained in drm1/2 but dependent on MET1. CMT3 depletion removes most of the CHG marks. (TIF) [file pone.0105338.s002.tif]

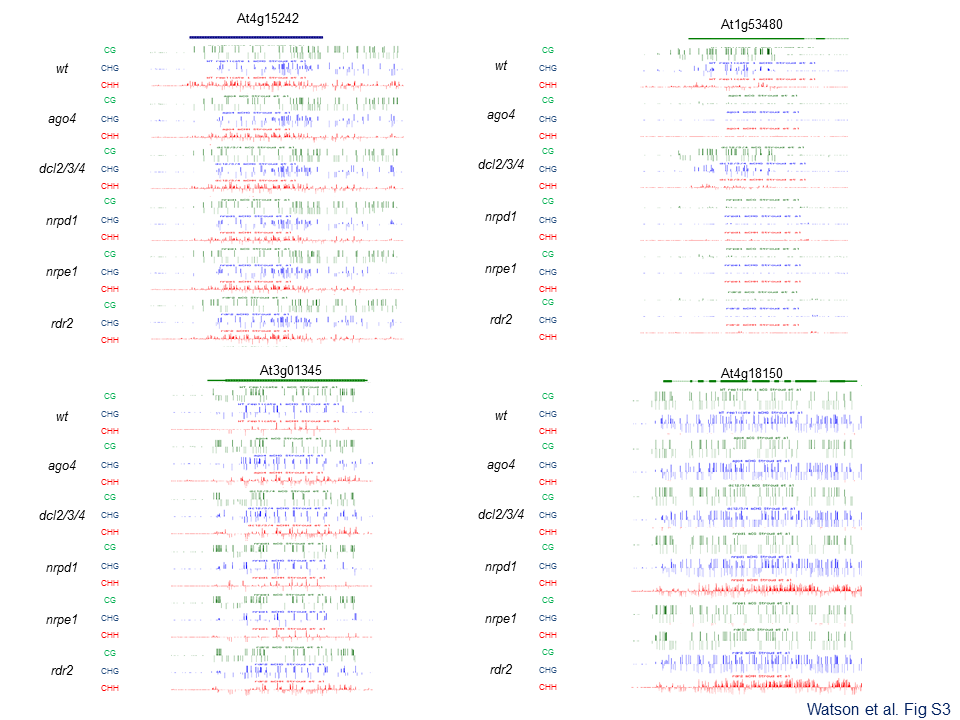

Supplement: Figure S3 — Methylation profile of four loci with dense DNA methylation in Columbia wildtype and in RdRM pathway mutants ago4, dcl2/3/4, nrpd1, nrpe1 and rdr2 [21] , accessed via http://genomes.mcdb.ucla.edu/AthBSseq/ ). With the exception of At1g53480, none of the genes change their methylation patterns in any of the mutants. (TIF) [file pone.0105338.s003.tif]

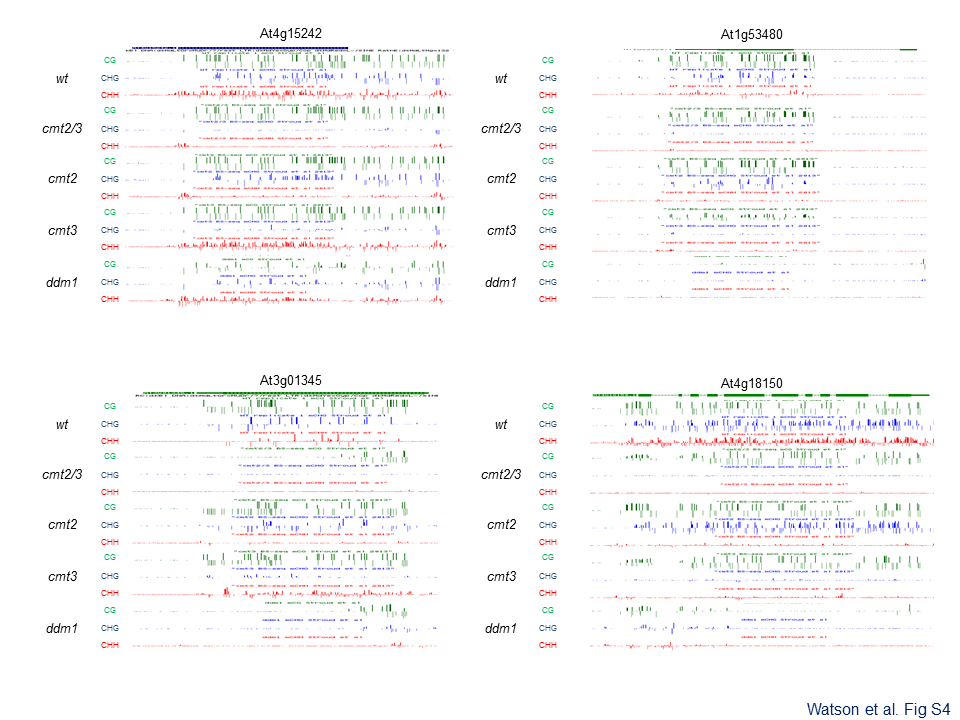

Supplement: Figure S4 — Methylation profile of four loci with dense DNA methylation in Columbia wildtype, cmt2/3, cmt2, cmt3 and ddm1 mutant lines [21] , accessed via http://genomes.mcdb.ucla.edu/AthBSseq/ ). In all genes, CHH methylation is significantly reduced in a cmt2 mutant. Loss of DDM1 causes loss of dense methylation in At1g53480 and partial loss of dense methylation in the other three lines. (TIF) [file pone.0105338.s004.tif]

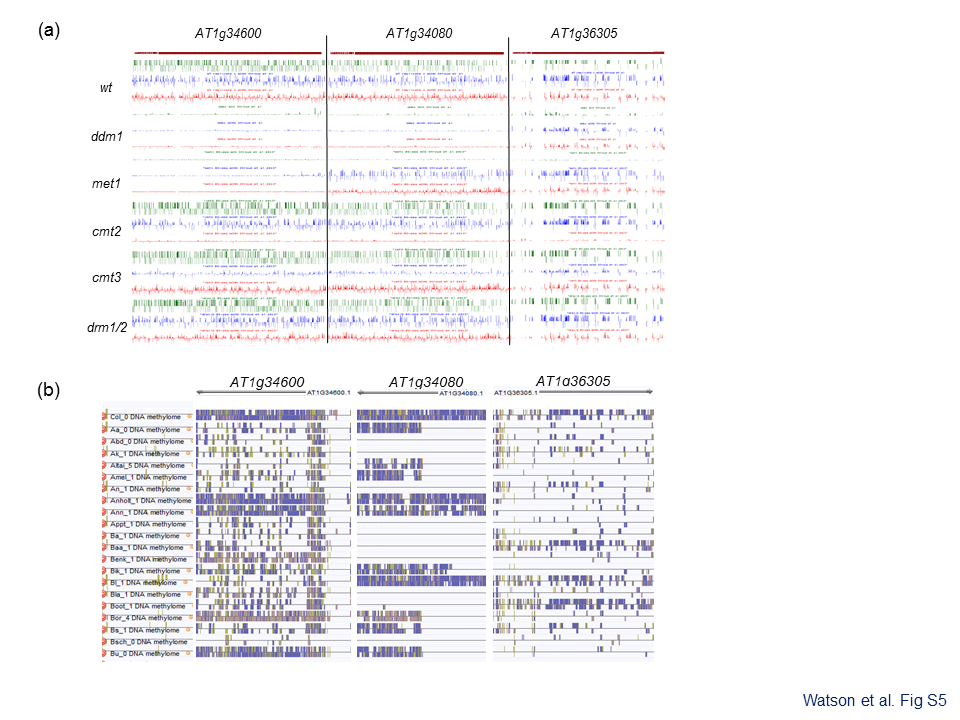

Supplement: Figure S5 — Methylation profiles of Gypsy elements in mutants and ecotypes. (a)Methylation profile of three Gypsy elements in Columbia wildtype, ddm1, met1, cmt2, cmt3 and drm1/2 mutant lines [21]. Methylation of the elements differs with respect to dependence on ddm1 and met1. A common feature of all elements is their dependence on CMT2 for CHH methylation and a reduction in CHG methylation in a cmt3 background. (b)Variable DNA methylation of the three Gypsy elements in different Arabidopsis ecotypes [18]. Irrespective of the methylation status, neither element is expressed. (TIF) [file pone.0105338.s005.tif]
